# *In Vitro α*-Glycosidase Inhibition and In Silico Studies of Flavonoids Isolated from *Pistacia integerrima* Stew ex Brandis

**DOI:** 10.1155/2022/9636436

**Published:** 2022-09-09

**Authors:** Hassan A. Hemeg, Abdur Rauf, Umer Rashid, Naveed Muhammad, Yahya S. Al-Awthan, Omar S. Bahattab, Mohammed A. Al-Duais, Syed Uzair Ali Shah

**Affiliations:** ^1^Department of Medical Laboratory Technology, College of Applied Medical Sciences, Taibah University, P.O. Box 344, Al-Madinah Al-Monawra 41411, Saudi Arabia; ^2^Department of Chemistry, University of Swabi, Swabi, Anbar, 23430 Khyber Pakhtunkhwa (KP), Pakistan; ^3^Department of Chemistry, COMSATS University Islamabad, Abbottabad Campus, 22060 Abbottabad, Pakistan; ^4^Department of Pharmacy, Abdul Wali Khan University, Mardan, Khyber Pakhtunkhwa (KP), Pakistan; ^5^Department of Biology, Faculty of Science, University of Tabuk, Tabuk 71421, Saudi Arabia; ^6^Department of Biology, Faculty of Science, Ibb University, Ibb 70270, Yemen; ^7^Department of Biochemistry, Faculty of Science, University of Tabuk, Tabuk 71421, Saudi Arabia; ^8^Biochemistry Unit, Chemistry Department, Faculty of Science, Ibb University, Ibb 70270, Yemen; ^9^Department of Pharmacy, University of Swabi, Swabi, Anbar, 23430 Khyber Pakhtunkhwa (KP), Pakistan

## Abstract

The galls of *Pistacia integerrima* are used in folk medicine for curing diabetes. The main aim of this study was the purification of flavonoids from galls of *P. integerrima.* The methanolic extract was subjected to column chromatographic analysis which afforded six flavonoids, namely, 3,5,7,4′-tetrahydroxy-flavanone (1), naringenin (2), 3,5,4′-trihydroxy,7-methoxy-flavanone (3), sakuranetin (4), spinacetin (5), and patuletin (6). These isolated compounds (1–6) were tested against *α*-glycosidase. The maximum antagonistic effect was noted against compound 6 (97.65%) followed by compound 5 (90.42%) and compound 1 (90.01%) at the same concentration (0.2 *μ*g). The inhibitory potential of all tested compounds was significant with a degree of variation from each other. Docking studies showed that all studied compounds interact with the active site residues *via* hydrogen bond interactions with hydroxyl groups, and thus, inhibition was enhanced. Hence, this finding would be the first screening of isolated flavonoids for *α*-glycosidase activity and with the mechanism of action. These flavonoids should be further investigated as candidate drugs for combating diabetes mellitus.

## 1. Introduction

*Pistacia integerrima* belongs to the family Anacardiaceae which is also commonly called zebrawood. It is a dioecious tree native to Asia and commonly found in Pakistan, Afghanistan, India, and West Himalayas. It grows at an altitude of 800–1900 m [1]. It comprises pinnately leaves and a single stem with several breaches. Its flowers are reddish in color and organized in panicles. It is distinct owing to galls on the petioles and leaves, which are used in various traditional systems and considered storehouses of bioactive compounds [2]. Its common traditional applications included appetite, phthisis, coughs, dyspeptic vomiting, dysentery, and asthma [3–6]. Several classes of compounds such as flavonoids, triterpenoids, sterols, and phenolic compounds are reported from various parts of *Pistacia integerrima* [7], including *β*-sitosterol, *β*-stigmasterol, pistagremic acid, naringenin, 3,5,7,4-tetrahydroxy flavanone [8], hydroxydecanyl arachidate, *n*-octadecan-9,11-diol-7-one, and pisticialanstenoic acid [9]. Various parts of *Pistacia integerrima* are explored for several biological activities such as antioxidant [7], phosphodiesterase inhibition [8], anticholine esterase activity [10], analgesic, anti-inflammatory [11, 12], muscle relaxant, gastrointestinal, antiemetic, antipyretic, and antidiarrheal [13–15]. The purpose of the present research was the isolation of the bioactive compounds from the methanolic extract of galls of *P. integerrima* and evaluation of *in vitro α*-glycosidase inhibition by methanolic extract/compounds, which is further investigated by docking studies of the isolated compounds at the molecular level.

## 2. Materials and Methods

### 2.1. Plant Collection and Processing

The galls of *P. integerrima* were obtained from various regions of Peshawar, Khyber Pakhtunkhwa, Pakistan. The plant specimen was identified by Dr. Muhammad Ilyas Department of Botany, University of Swabi, KP, Pakistan. The voucher specimen no. UOS/Bot-102 was kept in the herbarium of the Department of Botany University of Swabi, KP, Pakistan.

### 2.2. Extraction and Fractionation

**Figure 1:**
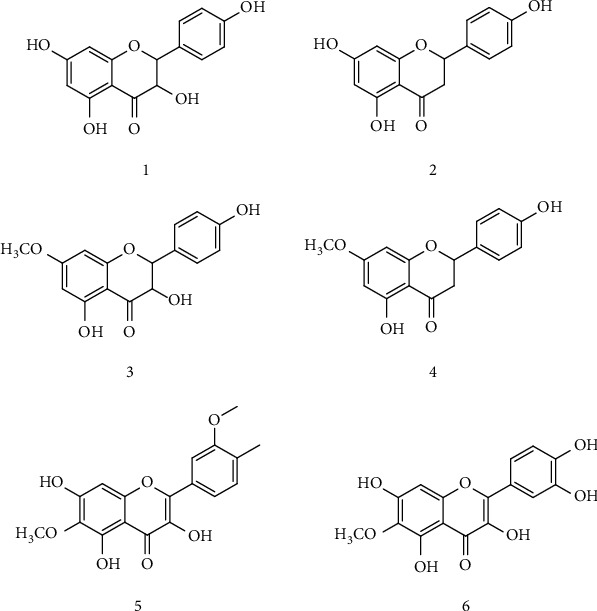
Chemical structures of isolated flavonoids (**1**–**6**) from *Pistacia integerrima.*

The galls were washed with water to remove the dust and dried under shade for 20 days. The dried galls (6.98 kg) were subjected to cold extraction with methanol (70 L) for 17 days. This extraction with methanol was done in triplicate. The methanolic extract (ME) obtained was filtered with filter paper and concentrated at low temperature and pressure to get the methanolic extract (ME) of galls (yield 134.76 g/6.98 kg of galls). The ME (6.98 kg) was further fractionated by liquid-liquid partitions using n-hexane, chloroform, and ethyl acetate which gives n-hexane soluble fraction (40.8 g), chloroform soluble fraction (79.43 g), ethyl acetate soluble fraction (65.65 g), and residue fraction (ME), respectively. The ethyl acetate fraction (26 g) was subjected to column chromatographic analysis over silica gel, then the column was eluted with chloroform and methanol (100 : 0 → 0 : 90). The subfractions obtained were assessed to a repeated chromatographic analysis by using chloroform and methanol (100 : 0 → 0 : 88) which afforded compounds (1–6; Figure 1). The chemical structures of all isolated compounds were identified by comparing the physical and spectral data with reported data [16].

### 2.3. *In Vitro α*-Glycosidase Assay

The isolated flavonoids (1–6) from *Pistacia integerrima* were assessed for *α*-glycosidase inhibitory potential as per reported methods [17, 18]. The acetone powder (intestine of rat) was mixed in normal saline at the ratio of 100 : 1 (w/v). The mixture was properly sonicated, and the upper layer was taken as a source of *α*-glycosidase. After the above treatment, 10 mL of tested samples was taken in DMSO and 100 mM phosphate buffer at pH of 6.8 in a 96-well plate and hatched out in 50 mL of intestinal *α*-glycosidase for a duration of five minutes before 50 mL substrate (5 mM, p-nitrophenyl-a-D-glucopyranoside agreed in the alike buffer) was included. Singular spaces for the test samples were fixed up to exact foundation absorbance where the substrate was altered with 50 mL of buffer. The control sample controlled 10 mL DMSO alongside test samples. The percent activity was calculated through the following equation.

(1) $$\begin{array}{c} \left[1-B/A\right]\times 100, \end{array}$$

where *A* is the absorbance of the control (DMSO) and *B* is the absorbance of the tested compounds.

### 2.4. Docking Studies

Docking studies of six flavonoids, namely, 3,5,7,4′-tetrahydroxy-flavanone (1), naringenin (2), 3,5,4′-trihydroxy,7-methoxy-flavanone (3), sakuranetin (4), spinacetin (5), and patuletin (6), were performed on the homology-modeled *α*-glycosidase already reported by our research group [19]. MOE 2016 version was used for this purpose. After preparing the constructed and validated homology enzyme, we determined the binding site of the enzyme [19–22]. For this purpose, a site finder was used. We selected the longest chain with 167 amino acid residues including three key residues of catalytic triad, i.e., Asp214, Glu276, and Asp349) [19]. For the validation of the docking protocol, we selected and docked standard drug acarbose and five randomly selected *α*-glycosidase inhibitors of natural as well as synthetic origin from the literature. Results of binding orientation and interaction by using all the parameters of methods and scoring functions at placement and refinement stages were analyzed to get reasonable performance. Finally, we selected a triangle matcher (at placement stage), London dG (scoring function), and GBVI/WA dG as the final score for docking studies. After docking, an analysis of the interaction of the ligand-enzyme complex was carried out by using the MOE interaction plot option.

### 2.5. Statistical Analysis

The results obtained were expressed as mean ± S.E.M. For statistical analysis, ANOVA was followed by post hoc Dunnett's test for multiple comparisons. In some cases, one sample *t*-test was used to evaluate significance against the hypothetical zero value. Values were considered to be significant at *P* ≤ 0.05.

## 3. Results

### 3.1. *α*-Glycosidase Inhibition

The inhibitory potential of isolated compounds against *α*-glycosidase is presented in Table 1. The results were presented as % inhibition and IC_50_ values. The maximum percent antagonist effect was noted against compound 6 (97.65%) followed by compound 5 (92.42%) and compound 1 (90.01) at the same concentrations (0.2 *μ*g). The inhibitory potential of all tested compounds was significant with a degree of variation from each other.

### 3.2. Docking Studies

**Figure 2:**
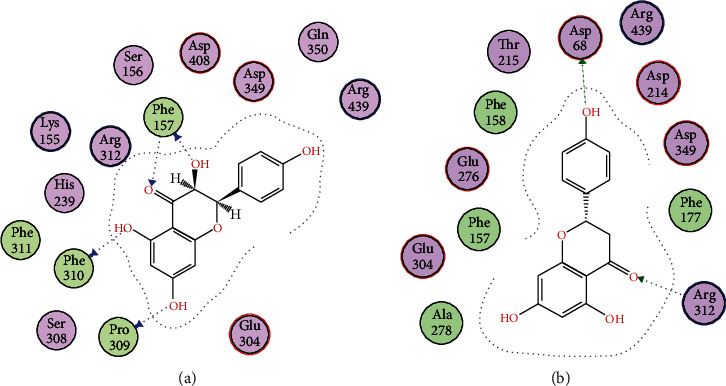
(a, b) 2D interaction plots of isolated compounds 1 and 2, respectively, into the binding site of homology-modeled *α*-glycosidase.

**Figure 3:**
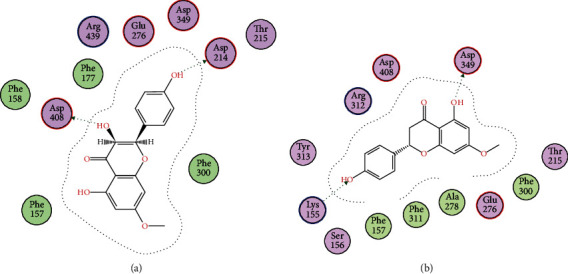
(a, b) 2D interaction plots of isolated compounds 1 and 2, respectively, into the binding site of homology modeled *α*-glycosidase.

**Figure 4:**
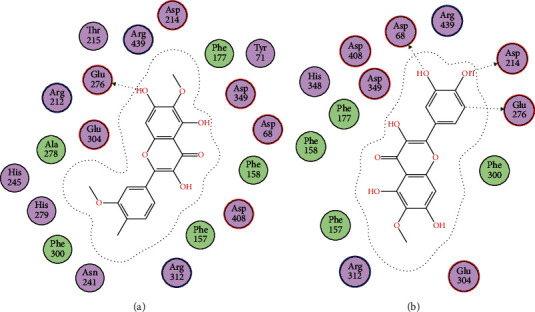
(a, b) 2D interaction plots of isolated compounds 1 and 2, respectively, into the binding site of homology-modeled *α*-glycosidase.

Docking studies of all the compounds were performed on the homology-modeled *α*-glycosidase already reported by our research group. The obtained two-dimensional interaction plots of all the compounds were analyzed. All the studied compounds are involved in the hydrogen bond interactions with the key amino acid residues in the active site *via* their hydroxyl and carbonyl oxygen. Compound 1 forms bifurcated hydrogen bonds with the carbonyl oxygen and hydroxyl group. Pro309 and Pro310 also formed hydrogen bond interactions with hydroxyl groups (Figure 2(a)). Compound 2 forms hydrogen bond interactions with Asp68 and Arg312. Compounds 3, 5, and 6 are oriented towards the catalytic triad and established hydrogen bond interactions with Asp214 and Glu276 (Figures 2(a), 3(a), and 4(a) and 4(b)), while compound 4 forms hydrogen bond interactions with Lys155 and Asp349 (Figure 3(b)).

## 4. Discussion

Natural products are the potential source of bioactive molecules with the best therapeutic effect against various ailments [23–25]. Various classes of phytochemicals such as flavonoids, triterpenes, phenolic compounds, and saponins isolated have been reported from *Pistacia integerrima.* These phytochemicals were reported previously for antioxidant, anti-inflammatory, analgesic, and antipyretic activity [26]. In the current advanced scientific era, various drug molecules have roots in natural products. Therefore, the screening of natural products is essential for the discovery of safe, effective, and economical therapeutic agents. In the current research work, the six isolated flavonoids from galls of *Pistacia integerrima* were screened for *α*-glycosidase inhibition. *Pistacia integerrima* Stewart is one of the best multimedicinal potential plants and used for various purposes such as antidiabetic, analgesic, anti-inflammatory, and liver protection [11–13]. Keeping in view the antidiabetic potential of this plant, the isolated constituents were tested against a potential target enzyme (*α*-glucosidase) of hypoglycemics with the best hope to find a new, safe, effective, and economical molecule. Hyperglycemic condition is one of the chronic problems that require lifelong management. The currently available drugs in the market are associated with various adverse drug reactions, and most of them have poor efficacy [27]. Due to this problem, the patient regularly changes the antidiabetic class for the best performance. Diabetes mellitus is induced through various mechanisms involving different enzymes like *α*-glucosidase [28]. These are located on the brush border of the small intestine and act upon the *α*-(1-4) glycoside linkage of carbohydrates such as starch and glycogen, thereby converting them into monosaccharides. This hydrolysis leads to a hyperglycemic condition known as diabetes mellitus; therefore, the antagonistic molecules for *α*-glucosidase are considered antidiabetics [29]. The well-known *α*-glucosidase inhibitors are acarbose and miglitol. Acarbose is used for the treatment of DM-II and prediabetes in China and Canada. Our tested compounds (**1**–**6**) isolated from galls of *Pistacia integerrima* significantly inhibited the alpha-glycosidase with variable potential and could be further evaluated for antidiabetic effects.

For a detailed understanding of the inhibition of *α*-glycosidase at the molecular level, the isolated compounds (**1**–**6**) were evaluated in docking studies against the active site of *α*-glycosidase. The presence of aromatic rings and hydroxyl groups were considered essential for various *α*-glycosidase inhibitors [30]. In docking simulations, all of the studied compounds interact with the active-site residues *via* hydrogen bond interactions with these hydroxyl groups and thus inhibition was enhanced. Compound **1** contains four hydroxyl groups and is capable of forming three hydrogen bond interactions with the active site of *α*-glycosidase, thereby supporting the potent *in vitro* inhibition of *α*-glycosidase in our data. The *in vitro* inhibition and in silico data of these isolated compounds indicate that these selected natural products might be used for further exploration with the best hope of finding new, effective, and safe candidate antidiabetics.

## 5. Conclusion

The present study concluded that the methanolic extract of galls of *Pistacia integerrima* inhibited alpha-glycosidase and led to the isolation of six compounds (**1**–**6**). All the isolated compounds inhibited alpha-glycosidase with variable potency. The docking studies further validated the interaction of the compounds with the active site of alpha-glycosidase. Thus, our data experimentally validated the folk use of *P. integerrima* as antidiabetic and the isolated compounds might be further evaluated as candidate antidiabetics. The limitation of the present study is the lack of evaluation of isolate compounds in the *in vivo* antidiabetic animal models for the exploration of the exact mechanism of action.

## Figures and Tables

**Table 1:** *α*-Glycosidase inhibition of methanol fraction and isolated flavonoids (**1**–**6**) from *Pistacia integerrima.*

Tested samples	Concentrations (*μ*g)	% inhibition	IC_50_ ± SEM (*μ*M)
Methanolic extract	0.2	87.43	128.87 ± 1.98
Compound 1	0.2	90.01	183.23 ± 1.22
Compound 2	0.2	79.54	754.23 ± 1.76
Compound 3	0.2	88.43	790.01 ± 2.31
Compound 4	0.2	73.98	287.34 ± 2.09
Compound 5	0.2	92.43	826.43 ± 1.87
Compound 6	0.2	97.65	743.12 ± 1.32
Standard (acarbose)	0.2	90.42	841.03 ± 1.34

## Data Availability

The data associated with this study to support the main finding of this paper will be available from the corresponding authors upon request.

## Supplementary Materials

Supplementary Materials The supporting information related to this manuscript is available online free of charge (See supplementary file). Scheme **1** and **2** for isolation of flavonoids isolated from *Pistacia integerrima.* (PDF).
