# Supplementary material for: In Vitro α-Glycosidase Inhibition and In Silico Studies of Flavonoids Isolated from Pistacia integerrima Stew ex Brandis
Source: Biomed Res Int. 2022 Sep 9;2022:9636436. doi: 10.1155/2022/9636436 (PMC9481312; doi:10.1155/2022/9636436)
Supplement: Supplementary Materials — The supporting information related to this manuscript is available online free of charge (See supplementary file). Scheme 1 and 2 for isolation of flavonoids isolated from Pistacia integerrima. (PDF). [file 9636436.f1.docx]

**In vitro α-glycosidase inhibition and in silico studies of Flavonoids isolated from *Pistacia integerrima* Stew ex Brandis**

Hassan A. Hemeg^1^, Abdur Rauf^2*^, Umer Rashid^3^, Naveed Muhammad^4^, Yahya S. Al-Awthan^5^, Omar Bahattab^5^, Mohammed A. Al-Duais^6,7,^ Syed Uzair Ali Shah^8^

^1^Department of Medical Laboratory Technology, College of Applied Medical Sciences, Taibah University, P.O. Box 344, Al-Madinah Al-Monawra 41411, Saudi Arabia.

^2^Department of Chemistry, University of Swabi, Swabi, Anbar, 23430, Khyber Pakhtunkhwa (KP), Pakistan.

^3^Department of Chemistry, COMSATS University Islamabad, Abbottabad Campus, 22060 Abbottabad, Pakistan

^4^Department of Pharmacy, Abdul Wali Khan University, Mardan, Khyber Pakhtunkhwa (KP), Pakistan.

^5^Department of Biology, Faculty of Science, University of Tabuk, Tabuk, Saudi Arabia.

^6^Department of Biochemistry, Faculty of Science, University of Tabuk, Tabuk, Saudi Arabia.

^7^Biochemistry Unit, Chemistry Department, Faculty of Science, Ibb University, Ibb, Yemen

^8^Department of Pharmacy, University of Swabi, Swabi, Anbar, 23430, Khyber Pakhtunkhwa (KP), Pakistan

**Corresponding author**

**Abdur Rauf**- Department of Chemistry, University of Swabi, Swabi, Anbar, 23430, Khyber Pakhtunkhwa (KP), Pakistan; [abdurrauf@uoswabi.edu.pk](mailto:abdurrauf@uoswabi.edu.pk)

**Scheme S1:** The extraction and fractionation of *Pistacia integerrima.*

Scheme S2: The isolation of flavonoids from ethyl acetate fraction of *Pistacia integerrima.*
